# Serum miR-22 as potential non-invasive predictor of poor clinical outcome in newly diagnosed, uniformly treated patients with diffuse large B-cell lymphoma: an explorative pilot study

**DOI:** 10.1186/s13046-018-0768-5

**Published:** 2018-05-02

**Authors:** Francesco Marchesi, Giulia Regazzo, Francesca Palombi, Irene Terrenato, Andrea Sacconi, Manuela Spagnuolo, Sara Donzelli, Mirella Marino, Cristiana Ercolani, Anna Di Benedetto, Giovanni Blandino, Gennaro Ciliberto, Andrea Mengarelli, Maria Giulia Rizzo

**Affiliations:** 1 Department of Clinical and Experimental Oncology, Hematology and Stem Cell Transplant Unit, IRCCS Regina Elena National Cancer Institute, Via Elio Chianesi 53, 00144 Rome, Italy; 2 Department of Research, Advanced Diagnostics and Technological Innovation, Genomic and Epigenetic Unit, Translational Research Area, IRCCS Regina Elena National Cancer Institute, Via Elio Chianesi 53, 00144 Rome, Italy; 3 Biostatistical Unit, IRCCS Regina Elena National Cancer Institute, Rome, Italy; 4 Department of Research, Advanced Diagnostics and Technological Innovation, Pathology Unit, IRCCS Regina Elena National Cancer Institute, Rome, Italy; 5 Scientific Direction; IRCCS Regina Elena National Cancer Institute, Rome, Italy

**Keywords:** DLBCL, Biomarkers, Circulating microRNA, miR-22, Let-7c cluster

## Abstract

**Background:**

Diffuse large B-cell lymphoma (DLBCL) is a heterogeneous group of tumors, with aggressive clinical course that renders prognostication and choice of treatment strategy difficult. Chemo-immunotherapy with rituximab, cyclophosphamide, doxorubicin, vincristine, prednisone (R-CHOP) is the current first-line treatment.

MicroRNAs (miRNAs) are under investigation as novel diagnostic and prognostic biomarkers in several malignancies, including malignant lymphomas. While tissue miRNAs in DLBCL patients have been extensively studied as biomarkers, only few reports to date have evaluated the role of circulating/serum miRNAs as potential prognostic factors.

Here circulating/serum miRNAs, including miR-22, were investigated as potential non-invasive biomarkers, with the aim of a better prognostic stratification of DLBCL patients.

**Methods:**

MiRNAs were selected by global expression profile of serum miRNAs of DLBCL patients, The Cancer Genome Atlas (TCGA) analysis and literature research.

Serum and tissues miRNA expression profile in de novo DLBCL patients, consecutively enrolled for this study, were detected by quantitative real-time polymerase chain reaction. Relative expression was calculated using the comparative Ct method. Statistical significance was determined using the Mann-Whitney rank sum and Fisher’s exact test. Survival analysis was conducted through the use of Kaplan-Meier method. Spearman’s Rho was applied to study the correlation between miRNA distributions and days to first relapse.

Experimentally validated miRNA-target interactions were assessed by miRTarBase database. Negative miRNA-mRNA correlation was evaluated in TCGA DLBCL dataset. Pathway analysis was performed by the functional annotation clustering DAVID tool.

**Results:**

We showed a significant modulation of serum miR-22 after R-CHOP treatment compared with basal values but no difference between baseline serum miRNAs values of DLBCL patients and healthy controls. High expression level of serum miR-22 in DLBCL at diagnosis (*n* = 36) is associated with a worse PFS and is independent of the currently used clinical prognostic index. Integrative and pathways analysis of miR-22 identified target genes involved in different important pathways such as p53 signaling.

**Conclusions:**

Our data suggest that miR-22 is of potential interest as non-invasive biomarker to predict clinical outcome in DLBCL patients. Characterization of miR-22 pathways can pave the way to the development of targeted therapy approaches for specific subgroups of DLBCL patients.

**Electronic supplementary material:**

The online version of this article (10.1186/s13046-018-0768-5) contains supplementary material, which is available to authorized users.

## Background

Diffuse large B-cell lymphoma (DLBCL), the most common high-grade non-Hodgkin lymphoma, is a heterogeneous group of tumors with aggressive clinical course [1]. Their heterogeneity makes prognostication and choice of treatment strategy difficult [2]. Immunochemotherapy with rituximab, cyclophosphamide, doxorubicin, vincristine, prednisone (R-CHOP) is the current first-line treatment [3]. However, with this therapeutic approach up to 40% of patients experience early treatment failure or relapse after initial response [4]. Baseline prognostic stratification is widely based on clinical indexes, such as the International Prognostic Index IPI; [5].

To date, some biological factors have been evaluated for their potential prognostic relevance in DLBCL [6]. In particular, different DLBCL subtypes, associated with distinct cells of origin and clinical outcomes, have been determined through gene expression profiling [7, 8] or immunohistochemistry (IHC) based algorithms, the most widely used of which is the Hans algorithm [9], that permits to identify two different subtypes of DLBCL despite some limitations in reproducibility. Moreover, Bcl-2, Bcl-6 and c-Myc aberrations have a potential role as prognostic markers of poor clinical outcome [10, 11], but they are applicable to a limited number of patients and not yet routinely used in clinical practice. Thus, DLBCL heterogeneity and the current lack of reliable predictors have prompted investigations for new biomarkers that can accurately predict survival [12].

Recently, body fluids have emerged as an important source of information in several pathologies, thus representing minimally invasive methods for precision diagnostics, prognostic stratification and treatment assessment. Despite some data have been recently published about cell-free DNA (cfDNA) monitoring in peripheral blood of DLBCL patients [13–15], so far few studies have highlighted the role of circulating microRNAs (miRNAs) in this setting. MiRNAs are under investigation as novel diagnostic and prognostic biomarkers in several malignancies [16, 17], including malignant lymphomas [18–22]. However, while tissue miRNAs in DLBCL patients have been extensively studied as novel diagnostic and prognostic biomarkers [18, 23–27], only few reports to date have evaluated the role of circulating/serum miRNAs as potential prognostic factors. In particular, high expression of miR-21 in malignant tissue and in blood has been associated with DLBCL diagnosis. [28–30], while other studies report an association with specific miRNA signatures and response to therapy [31–33].

## Methods

### Study design, patients and control subjects

This was a prospective, observational, non-interventional study on a cohort of newly-diagnosed de novo DLBCL adult patients consecutively enrolled at our department of Hematology and Stem Cell Transplant Unit (Regina Elena National Cancer Institute, IRE) and uniformly treated with six courses of R-CHOP every 21 days (rituximab 375 mg/m^2^ day 1, cyclophosphamide 750 mg/m^2^ day 1, vincristine 1.4 mg/m^2^ day 1, doxorubicin 50 mg/m^2^ day 1, prednisone 100 mg days 1–5), followed by two adjunctive doses of rituximab. The study was approved by the Institutional Ethical Committee of IRE (protocol number: RS 831/16); all patients and healthy subjects signed an informed consent before inclusion and were treated according to ethical and legal standards adopted by the Declaration of Helsinki. Histological criteria for diagnosis and classification of DLBCL are those of the World Health Organization (WHO) classification [34].

Patients were excluded if they presented a DLBCL transformed by a previous indolent lymphoma, concomitant active cancers, others life-threatening conditions that could compromise clinical outcome or HIV seropositivity. Serum samples of patients were collected after informed consent at diagnosis and then after 30–45 days from the end of the last treatment course at the moment of response assessment. All patients were evaluated for clinical and biological prognostic factors. Clinical assessment was performed by Ann Arbor stage and IPI evaluation. Cell of origin was evaluated by IHC using Hans algorithm, dividing immunophenotype pattern of patients in Germinal-Center (GC) vs non Germinal Center (non-GC) [9]. Formalin-fixed and paraffin-embedded (FFPE) tumor sections at diagnosis were analyzed for Bcl2, Bcl6 and c-Myc rearrangements by Fluorescent In Situ Hybridization (FISH), in cases of very high (> 80%) ki67 expression. Cases with concomitant c-Myc and Blc2 or Bcl6 rearrangements were defined as “double hit” lymphomas. Baseline disease staging and treatment response assessment were determined according to the Lugano recommendations for initial evaluation staging and response assessment of non-Hodgkin lymphoma [35].

Healthy controls were recruited at the same Institute from individuals seeking a routine health checkup and with no evidence of disease and with age-, gender- and ethnicity-matched to the patients.

### Study objectives

The objectives of the study were: *i)* to evaluate the difference in miRNAs expression at diagnosis and after R-CHOP therapy in serum of DLBCL patients; *ii)* to evaluate the correlation between circulating miRNA and response to treatment; *iii*) to find a specific miRNA signature significantly related to clinical outcome of patients in terms of PFS; *iv*) to investigate the correlation between circulating miRNAs and other baseline clinical-biological factors [gender, median age, Ann Arbor stage, IPI, lactate dehydrogenase (LDH) value, cell of origin, presence of Bcl2, Bcl6 and Myc rearrangements].

### Microarray

RNA from serum of DLBCL patients at diagnosis and post-treatment was extracted using the miRNeasy Mini kit (Qiagen, Hilden, Germany) following the manufacturer’s instructions. Concentration and purity of total RNA were assessed using a Nanodrop TM 1000 spectrophotometer (Nanodrop Technologies, Wilmington, DE, USA).

Total RNA (200 ng) was labeled and hybridized to Human miRNA Microarrays V19 (Agilent) for 2006 human miRNAs, using the miRNA Complete Labeling and Hyb kit to generate fluorescently labeled miRNAs.

### Sample processing and RNA extraction

Sampling method was consistent throughout the study to minimize any pre-analytical variables. Blood samples of DLBCL patients were collected at diagnosis and after R-CHOP treatment in BD Vacutainer serum tubes using a 21-gauge needle. The samples were kept at room temperature (RT) for 30–60 min and then centrifuged at RT for 20 min at 1100×g, the supernatant was further centrifuged for 5 min at 1300×g. The serum transferred into sterile cryovials was aliquoted and stored at − 80 °C until further analysis.

RNA was extracted from 200 μl of serum and purified using miRCURY RNA Isolation Kit – Biofluids (Exiqon #300112 Vedbaek- Denmark) in accordance with manufacturer’s instructions. For a precise and sensitive quantitation of total microRNAs concentration Qubit microRNA Assay Kits (Life Technologies #Q32880) was used on a Quibit 3.0 Fluorometer (Life Technologies-ThermoFisher, Waltham, MA U.S.A). There is considerable sample-to-sample variability in both protein and lipid content of plasma and serum samples, which could affect efficiency of RNA extraction, and could introduce potential inhibitors of PCR [36]. In order to minimize the technical variation between replicates in down-stream PCR analysis we added, for all isolations, spike-in non-human synthetic miRNAs (RNA spike-in mix: UniSp2, UniSp4 and UniSp5; Exiqon #203203) to the respective lysis/denaturant buffer before combining with serum. To avoid DNA contamination, all samples were subjected to on-column rDNase treatment in accordance with manufacturer’s instructions. After extraction RNA was eluted in 50 μl RNase-free water. Furthermore, determination of RNA yield is usually not possible by spectrophotometric reading; therefore, we used RNA amounts based on starting volume in the PCR reaction as a measure, combined with subsequent quantification of spike-ins. RNA from FFPE samples (*n* = 10) were extracted using AllPrepDNA/RNA FFPE Kit (Qiagen, Venlo, Netherlands) in accordance with manufacturer’s instructions. Total tissue RNA, eluted in RNase-free water, was quantified with the NanoDrop ND-1000 spectrophotometer (ThermoFisher Scientific, Wilmington, DE U.S.A.).

### Reverse transcription and quantitative real-time -PCR (qRT-PCR)

Quantification of the mature circulating and tissue miRNAs were performed by a miRNA-specific LNA™-based system using SYBR® Green (miRCURY LNA™ Universal RT microRNA PCR; Exiqon # 203301, Vedbaek- Denmark) as described [17].

First-strand cDNA was synthesized from 4 μl of each serum RNA sample or 20 ng of tissue RNA, using the Universal cDNA Synthesis kit II according to the Exiqon manufacturer’s protocols with any modifications (Exiqon, Vedbaek- Denmark). To control the potential presence of inhibitors and the quality of the cDNA synthesis reaction UniSp6 RNA Spike-in template was added to the Reverse Transcription mixture.

The cDNA template was diluted 40× in nuclease-free water and then amplified using microRNA-specific LNA™-enhanced forward and reverse primers. QRT-PCR was performed employing an ABI 7900 Real Time PCR System and SDS 2.2.2 software (Applied Biosystems, Foster City, CA). All reactions were performed in triplicate and for the background level a No Template Control was included in the study every time a new experiment was set-up. ROX passive reference dye was added in the diluted cDNA samples to obtain a robust read over the entire array of wells (ROX solution, Thermo Fisher Scientific, Waltham, MA USA). Expression data for miRNAs were analyzed calculating cycle threshold (Ct) values as well as standard deviations by means of comparative ΔCt method. The quantity of serum and tissue miRNAs was normalized as described below.

Established consensus house-keeping miRNAs for data normalization are lacking for serum miRNAs. Exiqon manufacturer’s protocols suggest miR-103-3p as a candidate endogenous reference gene but it showed high variation in our samples. Thus, in order to minimize variation in circulating miRNA recovery, retro-transcription and amplification efficiency, we normalized serum miRNA levels measuring the expression of the synthetic spiked-inUniSP2 (UniSP2 LNA control primer set UniRT, Exiqon#203950).

The expression levels of mature tissue miRNAs were normalized to the U6 snRNA (U6 snRNA LNA primer set UniRT Exiqon #203907).

As the major source of variation in plasma and serum miRNA expression patterns is potential cellular derived miRNA contamination including hemolysis [37] we screened selected samples for hemolysis analyzing the expression levels of miR-451, abundant in red cells, and miR-23a, unaffected by hemolysis, as suggested by Exiqon manufacturer’s protocols (samples with Ct miR-23a – Ct miR-451 ≥ 5 are considered hemolyzed).

### Data and the Cancer genome atlas (TCGA) analysis, bioinformatics

Scanning and image analysis were performed using the Agilent DNA Microarray Scanner (P/N G2565BA) equipped with extended dynamic range (XDR) software according to the Agilent miRNA Microarray System. Signals from miRNA’s arrays were verified for quality control and extracted by Agilent Feature Extraction 10.7.3.1 software. All values lower than 1 were considered below detection and thresholded to 1. The signal of each sample was z-score transformed. Bioinformatic analyses were performed by MATLAB (The MathWorks Inc.). Deregulation of miRNAs was assessed using a permutation test and a false discovery procedure was included for multiple comparisons [38]. Statistical significance was set to 5%. Unsupervised hierarchical clustering was performed to identify specific pattern of expression using the Euclidean distance metric.

A survival analysis was performed on a list of selected miRNAs.

The experimentally validated miRNA-target interactions database miRTarBase, release 7.0 [39] was used to select validated targets of specific miRNAs. A Negative miRNA-mRNA correlation was then evaluated in TCGA DLBCL dataset for each validated target.

The list of validated targets was filtered by considering those genes with a negative correlation coefficient (Spearman’s *R* < − 0.2, *p* < 0.05). A pathway analysis was conducted with the functional annotation clustering tool DAVID [40].

### Statistical analysis

Descriptive statistics were calculated for all the variable of interest to summarize patient’s characteristics. Due to the small sample size, the most suitable non parametric test was applied to evaluate the associations between variables. Spearman’s Rho was applied to study the correlation between miRNA distributions and days to first relapse. Survival analysis was conducted through the use of Kaplan-Meier method. Log-rank test was used to individuate potential differences between subgroups. PFS was defined as the time interval between the date of initial diagnosis and the date of disease progression or death from any cause, whichever occurred first. OS was defined as the time interval between the date of initial diagnosis and the date of death from any cause. In order to identify independent predictors of progression, Cox proportional hazard models were built. The related estimates were reported as Hazard Ratios (HR) and 95% Confidence Intervals (CI). A *p*-value ≤0.05 was considered statistically significant. All analyses were carried out with SPSS (version 21.0) statistical program (SPSS Inc., Chicago, IL, USA). Principal Component Analysis (PCA) was performed using MATLAB software (The MathWorks Inc.)

## Results

### Patients characteristics

From September 2015 to February 2017, a total of 36 newly diagnosed DLBCL patients were enrolled into the study. We were able to collect serum samples at diagnosis and after the end of treatment in 32 patients, whereas the remaining 4 cases did not result evaluable for post-treatment analysis (toxic deaths during treatment in three cases, loss of follow-up in one case). Baseline clinical and biological features and treatment response assessment of these 36 patients are shown in Table 1.

**Table 1:** General characteristics and baseline clinicopathological features of DLBCL patients

Variables		DLBCL *n* = 36 (%)
Age median (range)		62 (23–83)
Gender males/females		20/16
Ann-Arbore stage	I-II	9 (25)
	III-IV	27 (75)
IPI	Low (0–1)	8 (22)
	Medium (2–3)	15 (42)
	High (4–5)	13 (36)
LDH	Normal	18 (50)
	High	18 (50)
Ki-67	< 70%	15 (42)
	≥ 70%	21 (58)
Cell of origin	GC	22 (61)
	Non-GC	10 (28)
	NE	4 (11)
Translocations (§)	None	10 (28)
	Single	10 (28)
	Double hit	4 (11)
	NE	12 (33)
Response to treatment (^)	ORR	27 (84)
	CRR	24 (75)
	Primary refractory	5 (15)

*NE*: not evaluable; *ORR*: overall response rate, *CRR*: complete remission rate; *GC*: germinal-center;

(§) Translocations evaluated on BCL2, BCL6, and MYC loci

(^) Response to first-line treatment was assessed in 32 patients. Four patients were not evaluable for

treatment response assessment (early deaths *n* = 3, lost *n* = 1)

Out of 32 patients evaluable for treatment response, 27 (84%) achieved a response to first-line treatment (responders); 24 of them achieved a complete remission (CR). Five patients (15%) were primary refractory (non responders). In responder patients, a further program of standard follow-up according to current guidelines [5] was started and three of them experienced a disease relapse. Refractory/relapsed patients underwent a salvage treatment according to published guidelines [5] and to local policy.

### Selection of miRNAs as potential prognostic biomarkers for DLBCL patients

In order to select a panel of miRNAs whose expression level in serum might be related to the risk of disease recurrence and survival of the DLBCL patients, we performed (*i*) a global expression profile of serum miRNAs of a small cohort of DLBCL patients; (*ii*) a study based on data available on TCGA data portal [41] of DLBCL; (*iii*) an analysis of expression profiles of miRNAs selected from (*i*) and (*ii*) or described as associated with lymphoid malignancies by us (unpublished observation) or by previously published studies [18, 32, 42] in serum samples of patients enrolled into the study.

**Fig. 1:**
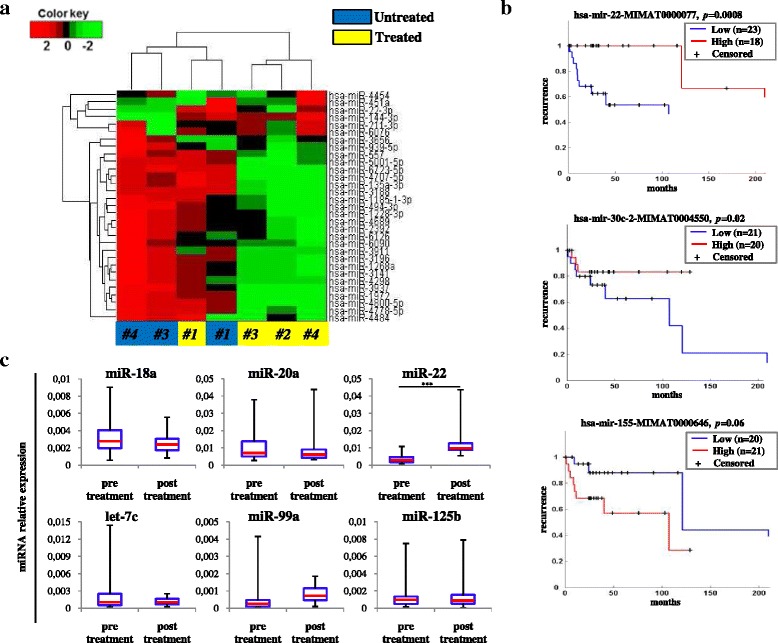
Selection of miRNAs to evaluate as prognostic biomarkers for DLBCL patients. **a** Heat-map of hierarchical clustering of 31 selected miRNAs on serum of DLBCL patients at diagnosis (untreated) and after R-CHOP treatment (treated). MiRNA expression levels are shown as colour variations. Higher and lower values are represented by red and green points, respectively. Pairwise distances between rows and between columns were computed by Euclide distance metric. **b** Kaplan-Meier PFS curves. Correlation between the indicated miRNAs and the rate of recurrence of DLBCL patients from TCGA data analysis. **c** Box-plot diagrams of relative miRNA expression levels, before/after treatment, in serum samples from DLBCL patients (*n* = 16), assessed by qRT–PCR. Box-plot diagrams of selected miRNA expression levels in pre-treatment and post-treatment serum samples from a small cohort of DLBCL patients (n = 16). Boxes define the 25^th^ and 75^th^ percentiles; the horizontal line into the boxes indicates the median, and bars define the minimum and maximum values. The expression levels of mature miRNAs were normalized to volume and UniSp2 spike-in RNA. Relative expression was calculated using the comparative ΔCt method. *p*-values (*** = *p* ≤ 0.001) were determined using the Mann–Whitney rank sum test

Our microarray analysis was performed in pre- and post-treatment serum samples derived from three DLBCL patients responding to therapy and one non-responding patient (samples #1, #3, #4 collected at diagnosis and #1, #2, #3, #4 after the end of R-CHOP treatment). Results showed that 153 serum miRNAs were expressed above background in at least 3 out of 7 samples analyzed. Interestingly, we found a striking difference in miRNA modulation upon treatment between responder and non responder patients. Due to the limited number of samples, in this analysis we did not focus on miRNA signature discriminating the group of responders versus the non responders but rather on miRNAs significantly modulated in DLBCL samples upon R-CHOP treatment. As shown in Fig. 1a, we found 31 miRNAs, including miR-22 (*p* < 0.05; permutation test), significantly modulated after R-CHOP in the group of responsive patients. In contrast, this miRNA subset did not show remarkable expression changes to first-line treatment in non responder patient #1. Moreover, for a comparison of levels of expression of serum miRNAs of our DLBCL patients with regard to tumor tissues we performed a study interrogating the TCGA database where we found available data relative to the miRNA expression levels in tumor tissue samples of 47 DLBCL patients. Kaplan Meier curves and log-rank test revealed a signature of 13 miRNAs with potential prognostic value. In particular, 3 miRNAs (miR-22-3p, miR-30c-2-3p, miR-155-5p) were linked to disease recurrence (Fig. 1b), whereas 10 others (miR-29c-3p, miR-132-3p, miR-140-5p, miR-142-5p, miR-146a-3p, miR-215-5p, miR-330-3p, miR-338-3p, miR-582-3p, miR-582-5p) were significantly related to OS (Additional file 1 Fig. S1). Since these are observational data, we could speculate that the disparity between the number of miRNAs associated to PFS and OS could be due to the relative small size of the sample available on TCGA for this pathology (47 patients). However, we cannot exclude that in a wider cohort of patients other miRNAs could be found associated with these variables.

Using RT-qPCR we profiled, in a small cohort (*n* = 16) of DLBCL patients, the expression of miR-22 found in both our previous analysis (Fig. 1a,b) as modulated in DLBCL patients as well as selected circulating miRNAs described as associated with lymphoid malignancies by us (the cluster of let-7c/miR-99a/−125b, unpublished results) [32] or by published studies (mir-18a, −20a) [18, 42] in serum samples collected before/after treatment. As shown in Fig. 1c, we found that in de novo DLBCL patients only serum miR-22 was significantly up-regulated (2.9 folds; *p* ≤ 0.001) in post-treatment samples compared with matched patients before treatment.

These data suggest that the serum miR-22 is of potential interest as non–invasive biomarker to predict therapeutic response in DLBCL patients.

### Expression level of serum miR-22 and let-7c/miR-99/−125b cluster in a cohort of DLBCL patients

**Fig. 2:**
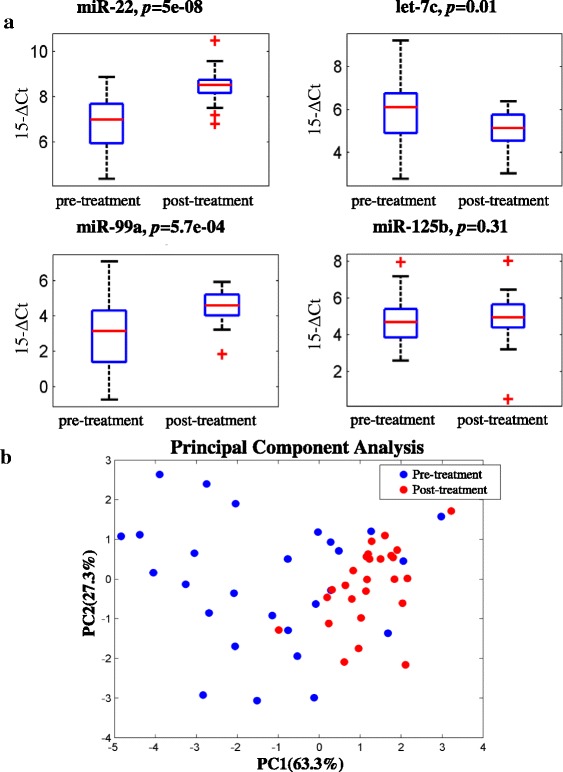
Expression levels and PCA analysis of serum miR-22 and let-7c/miR-99a/−125b cluster in a cohort of DLBCL patients. **a** Box-plot diagrams of relative miRNA expression levels in pre-treatment and post-treatment serum samples from DLBCL patients. Boxes define the 25^th^ and 75^th^ percentiles; the horizontal line into the boxes indicates the median, and bars define the minimum and maximum values. The expression levels of mature miRNAs were normalized to volume and UniSp2 spike-in RNA. *p*-values were determined using the Mann–Whitney rank sum test. **b** Principal Component Analysis plots for pre- and post-treatment samples considering the combination of miR-22, let7c and miR-99a serum levels

Expression profile of miR-22 and let-7c cluster were evaluated by qRT-PCR, as potential non-invasive prognostic biomarkers in the serum of 32 out of 36 DLBCL patients at diagnosis and after R-CHOP treatment (Table 1). According to first objective (*i*) our data showed a significant modulation of serum miR-22, let-7c and miR-99a after R-CHOP treatment compared with basal values (Fig. 2a). Since most patients of this cohort are responders to R-CHOP, the significance of these first data seems controversial. For this reason we evaluated if there was a general modulation of miRNAs expression upon treatment. To this end, we measured the total miRNAs concentration in a representative group of pre- and post-treatment RNA samples extracted from our cohort of DLBCL patient. We observed no significant difference in global miRNAs concentration between pre- and post-treatment samples (data not shown) thus suggesting that the modulation observed for miR-22, let-7c and miR-99a is specific for these miRNAs.

We have subsequently analyzed our data performing a Principal Component Analysis (PCA) (Fig. 2b) on pre- and post-treatment samples considering the combination of miR-22, let7c and miR-99a that are the miRNAs modulated upon treatment. As shown in Fig. 2, pre- and post-treatment populations are clearly separated based on miRNAs expression. Moreover, we observed that pre-treatment samples show more variability in miRNAs expression, while miRNA levels in post-treatment samples appear to be more homogeneous.

In addition, a comparison between baseline serum miRNAs values of DLBCL patients and healthy controls matched for age and gender was performed and we did not find any significant difference between the two groups suggesting no diagnostic value for the analyzed miRNAs in DLBCL (data not shown). Even if modulation of miR-22, let-7c and miR-99a before and after treatment could suggest a role of these serum miRNAs in response to treatment, according to objective (*ii*), our data did not show a statistically significant correlation between circulating miR-22, let-7c, miR-125b and miR-99a and response to first-line R-CHOP.

### Values of miR-22 at diagnosis significantly affect clinical outcome

**Fig. 3:**
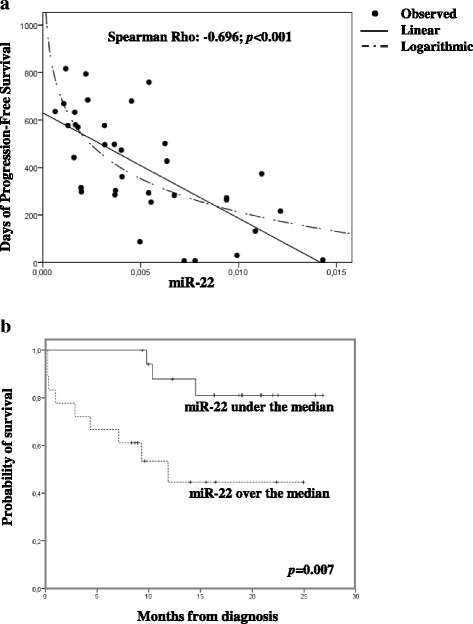
Expression of serum miR-22 is predictive of PFS in DLBCL patients.** a** Linear and logarithmic correlation between serum miR-22 values at diagnosis and days of PFS (Rho di Spearman = − 0.696; *p* < 0.001). **b** Kaplan–Meier curves for PFS in DLBCL patients with low (below median; solid line) and high (above median; dashed line) expression of miR-22. [*p*-value (log-rank test) = 0.007]

According to objective *(iii)*, we found a significant correlation between values of serum miR-22 at diagnosis and days of PFS (Rho di Spearman: − 0.696, *p* < 0.001; Fig. 3a); in other words, patients expressing higher values of serum miR-22 at diagnosis showed a worse clinical outcome in terms of PFS and had a higher risk of disease resistance or recurrence. Moreover, as shown in Fig. 3b, the expression of miR-22, analyzed as a dichotomous variable (above and below median serum level), was significantly correlated with PFS (median PFS of patients with miR-22 serum level above vs below the median: 12 months vs not reached; *p* = 0.007). On the contrary, expression of all members of let-7c cluster was not associated with PFS. Moreover, we examined whether miR-22 serum levels could be considered an independent prognostic factor. With Cox regression analysis, a median value of miR-22 above the median at diagnosis was found to be the only factor able to independently affect the probability of PFS in our cohort of patients (hazard ratio: 5.19, 95% CI: 1.38–19.56; *p* = 0.015, Table 2).

**Table 2:** Progression Free Survival (PFS) analysis by Cox regression results

Factor	HR	95% CI	*p*-value
miRNA-22 (above vs. below median value)	5.19	1.38–19.56	0.015
IPI			
Intermediate vs. low	1.03	0.19–5.62	0.977
High vs. intermediate-low	2.07	0.67–6.48	0.209
Cell of origin (non-GC vs. GC)	0.42	0.13–1.38	0.153
Double hit (yes vs. no) (^)	2.88	0.78–10.72	0.113

*HR:* hazard ratio; *CI:* confidence intervals*; IPI:* International Prognostic Index;

*GC*: germinal-center; *non-GC*: non germinal center

(^) Datum not available for all patients (see methods)

With respect to objective (*iv*), we did not find any significant correlation between circulating miRNAs expression and other clinical and biological factors (age, stage, performance status, LDH, Ki67, cell of origin, cytogenetics and extranodal involvement), with the only exception of let-7c (data not shown). In particular, no significant correlations between miR-22 values and other parameters were observed, whereas we found that patients expressing low levels of serum let-7c presented more frequently a high-risk IPI score (*p* = 0.032) and a Ki67 expression < 70% (*p* = 0.048).

### TCGA/DLBCL dataset: Integrative and pathways analysis of miR-22\mRNA

Since we found that only miR-22 at diagnosis significantly affects clinical outcome we performed a Spearman correlation on matched 47 miRNA\mRNA samples (miRNA-seq\RNA-seq) in TCGA DLBCL dataset for this miRNA. MiR-22 expression was correlated to specific targets, experimentally validated, selected by miRTarBase [39]. As shown in Table 3, a total of five validated target genes of miR-22 showing a negative correlation (Spearman’s *R* < − 0.2, *p* < 0.05) such as cyclin-dependent kinase 6 (*CDK6*) and cyclin-dependent kinase inhibitor 1A (*CDKN1A*, also referred to as p21) were obtained. The resulting gene list was also used for a pathway analysis with the functional annotation clustering tool DAVID. We found several interesting pathways significantly enriched in miR-22 targets (Table 4). Interestingly above all the key genes *CDK6* and *CDKN1A* were involved in different important pathways including p53 pathways [43]**.**

**Table 3:** miR-22 validated targets from TCGA data set anti-correlation analysis

MicroRNA	Gene	R-Spearman	*p*-value	PUBMED ID
hsa-miR-22-3p	CDK6	−0.351	0.016	20,371,350
hsa-miR-22-3p	CDKN1A	−0.433	0.003	23,582,783\21,572,407
hsa-miR-22-3p	LONP2	−0.312	0.033	23,824,327\27,418,678
hsa-miR-22-3p	TFRC	−0.303	0.039	19,135,902
hsa-miR-22-3p	ZNF460	−0.424	0.003	23,592,263

**Table 4:** Pathways significantly enriched in miR-22 targets

Term	Genes	*p*-value
hsa05214:Glioma	CDKN1A, CDK6	0.018
hsa04115:p53 signaling pathway	CDKN1A, CDK6	0.019
hsa05218:Melanoma	CDKN1A, CDK6	0.02
hsa05220:Chronic myeloid leukemia	CDKN1A, CDK6	0.02
hsa04066:HIF-1 signaling pathway	CDKN1A, TFRC	0.028
hsa04110:Cell cycle	CDKN1A, CDK6	0.035
hsa05161:Hepatitis B	CDKN1A, CDK6	0.041

## Discussion

DLBCL heterogeneity has prompted investigations for new biomarkers that can accurately predict survival. Several pivotal studies suggested the existence of potential prognostic biomarkers, specific pathogenetic pathways, and different mechanisms of therapy resistance among the subtypes [7, 8, 44]. Robust prognostic tools may allow stratification of treatment modalities avoiding exposure to unnecessary treatment toxicity or suboptimal therapy. Circulating miRNAs are very attractive candidates as non-invasive biomarkers for their high stability in serum/plasma, specificity, sensitivity and predictive power on disease stage and treatment response [45–48]. Moreover, miRNAs may be detected at low quantities, even at picogram concentrations [20] thus, their expression is likely to be more robust in the determination of cellular origin of multiple cancers [49].

On these bases, the main aim of this study was to find a specific miRNA signature significantly correlated with clinical outcome, in terms of PFS or response to treatment, and to other clinicopathological characteristics in a cohort of DLBCL patients treated with the current gold standard therapy R-CHOP. To this end, we analyzed the expression profile of some miRNAs, selected from our experimental data (miR-22 and the cluster of let-7c/miR-99a/−125b) or described as associated with lymphoid malignancies by previously published studies (miR-18a and miR-20a) [18, 42]. We found that miR-22 could have a potential role as new and non-invasive prognostic biomarker of clinical outcome in DLBCL patients uniformly treated con R-CHOP, since patients with higher serum miR-22 expression at diagnosis showed a strongly worse clinical outcome in terms of PFS; moreover, as far as correlation to treatment response is concerned, we observed a trend (even if not statistically significant) toward high serum miR-22 levels at diagnosis and a lower probability to achieve a response to R-CHOP (data not shown).

As far as miRNAs modulation upon treatment is concerned, from PCA analysis results we can hypothesize that miRNA variability before treatment is due to differences between patients (possibly discriminating their prognosis) while miRNA increase after treatment, more homogeneous among different patients, is a more generalized event that could be due to the therapy itself (a variable that is common to all patients) maybe reflecting the massive immune cells depletion that is a consequence of R-CHOP [50].

Our data seem to be promising given that the role of circulating miR-22 as independent prognostic marker of poor clinical outcome was confirmed by Cox regression analysis to be not influenced by other well-established prognostic factors, such as IPI, cell of origin and cytogenetics. However, we are aware that the present study has some limits due to the small number of patients enrolled so far, thus our multivariate analysis should be further confirmed in a larger cohort of DLBCL patients. Moreover, the median follow-up of enrolled patients was relatively short (18 months); and the evaluation of cell of origin in our patients cohort has been performed by using IHC and not by GEP, negatively affecting the real prognostic role of this biological parameter.

Our analysis of data available on TCGA database about miR-22 levels in tumor tissues from DLBCL patients displayed that lower miR-22 levels were associated with a worse prognosis.

In our study we attempted to perform a comparison of the miRNA expression patterns between serum and tissues to provide additional evidence supporting the use of serum miRNAs as reliable prognostic biomarkers. However, due to very low number of available specimens we did not find any significant correlation between tissue and serum values of selected miRNAs (data not shown). Although several studies demonstrate a direct correlation between serum and tumor tissue miRNA expression levels, suggesting that circulating miRNAs are a mirror of tissue miRNA levels, an inverse relationship between cell-free and cellular miRNAs has been already reported for different kind of tumor tissues and cell lines [51–53]. Thus, these observations might reflect a yet undefined molecular mechanism of selective secretion of miRNAs into the extracellular environment by tumor cells [52]. Furthermore, a complete knowledge of the molecular mechanisms governing miRNAs release from normal and tumor tissues is still lacking, and the debate about the relative contribution of organs and/or tissues to miRNAs in blood serum is still open [47, 54, 55]. Moreover, several suggested miRNA predictors obtained from tumor tissue specimens are not detected in serum by studies on the same kind of tumor, thus suggesting that the predictive role of serum miRNAs could be independent from tissue specimens [56] and references therein.

Moreover, we found that *CDKN1A* and *CDK6*, validated target genes of miR-22, are inversely correlated with miR-22 levels from TCGA data analysis. The gene encoding miR-22 resides on the short arm of chromosome 17, is ubiquitously expressed in various tissues [43, 57], and is highly conserved across many vertebrate species [57]. This level of conservation suggests that miR-22 plays a functional important role in life processes. Recently, miR-22 was found down-regulated in many cancers [43, 57] and has been assigned a role of tumor suppressor miRNA in advanced disease and metastasis in several cancers including lymphoma [57–60]. However, the role of miR-22 in lymphomagenesis still remains largely unknown. It has been also demonstrated that the normal function of this tumor suppressor miRNA is to down-regulate a number of putative oncogenes including validated miR-22 targets *MAX, MYCBP, HDAC4, HDAC6, CDK6, CDKN1A* and *NCoA1* [58]. In our study, we identified ten pathways significantly enriched with the miR-22 anticorrelated genes related to cell cycle, *CDK6* and *CDKN1A*. CDK6 is a member of the CDK family, which comprises heteromeric serine/threonine kinases that control progression and regulate mammalian cell division through the cell cycle in collaboration with their regulatory subunits, the cyclins [61, 62]. CDKN1A is a CDK inhibitor which physically interacts with, and inhibits, the activity of cyclin-CDK2, -CDK1, and -CDK4/6 complexes, preventing phosphorylation of critical cyclin-dependent kinase substrates thus functioning as a regulator of cell cycle progression during the G1 and S phases [63]. Interestingly, both *CDK6* and *CDKN1A* are involved in p53 signaling pathway, suggesting that the p53–miR-22–*CDK6* and/or *CDKN1A* axis could play a major regulatory role in the determination of p53-dependent apoptosis [61, 64, 65]. In particular, *CDKN1A* regulation by miR-22 has been shown to selectively determine the induction of p53-dependent apoptosis over cell cycle arrest acting as a molecular switch for the determination of p53-dependent cellular fate in response to various oncogenic stresses characterized by different damage intensity [65]. Furthermore, it has been demonstrated that miR-22 constitutes a feedback loop with c-myc and MYCBP and forms a regulatory loop in the phosphatase and tensin homolog–AKT pathway [66–68] another pathway that we found significantly enriched in miR-22 *CDKN1A* and *CDK6* targets. Thus, our integrative and pathways analysis of miR-22 suggest that this miRNA may play a critical role in DLBCL through regulating important pathways such as p53 signaling. *TP53* mutations analysis is essential to exclude that the worse clinical outcome of patients expressing higher values of serum miR-22 is due to these already know prognosticators in DLBCL [13]. To this regard, our unpublished observations on a limited cohort of DLBCL patients suggest that no correlation exists between *TP53* mutations and miR-22 expression levels in serum. Anyway this topic deserves to be further investigated in a larger number of samples.

## Conclusions

The identification of patient-specific miRNA expression profiles could be a useful tool to predict the response to standard chemo-immunotherapeutic treatment that would allow a “personalized medicine” approach for these patients, with potential clinical advantages deriving from the best possible therapy for that specific biological subset of patients. In addition, the hierarchical clustering of miRNAs and pathways, based on the levels of their interactions, can pave the way to the development of targeted therapy approaches for specific subgroups of DLBCL. Here, we show - for the first time to our knowledge - that the expression level of serum miR-22 in DLBCL is associated with survival and is independent of the currently used clinical prognostic index IPI. However, we are aware that prospective, large-scale, multicentre studies are necessary to confirm our results and the tumorigenic mechanisms of this miRNA in DLBCL warrants further investigations.

## Additional file

Additional file 1: **Fig. S1.** Kaplan-Meier Overall Survival curves. Correlation between the indicated miRNAs and the Overall Survival of 47 DLBCL patients from TCGA data analysis. (PDF 204 kb)

## Abbreviations

ABC: Activated B-cell
CDK6: Cyclin-dependent kinase 6
CDKN1A: Cyclin-dependent Kinase inhibitor 1A
cfDNA: Cell-free DNA; IRE, Regina Elena National Cancer Institute
CI: Confidence Interval
CR: Complete Remission
CRR: Complete Remission Rate
Ct: Cycle threshold
DLBCL: Diffuse large B-cell lymphoma
FFPE: Formalin-fixed and paraffin-embedded
FISH: Fluorescent In Situ Hybridization
GC: Germinal-Center
GEP: Gene Expression Profile
HR: Hazard Ratios
IHC: Immunohistochemestry
IPI: International Prognostic Index
IRE: Regina Elena National Cancer Institute
LDH: Lactate dehydrogenase
miRNA: microRNA
NE: Not evaluable
non-GC: Non Germinal Center
ORR: Overall Response Rate
OS: Overall Survival
PCA: Principal Component Analysis
PFS: Progression-Free Survival
qRT-PCR: Quantitative reverse-transcription polymerase chain reaction
R-CHOP: Rituximab, Cyclophosphamide, Doxorubicin, Vincristine, Prednisone
RT: Room temperature
TCGA: The Cancer Genome Atlas
WHO: World Health Organization
XDR: Extended dynamic range
