# Supplementary figures and images for: Serum miR-22 as potential non-invasive predictor of poor clinical outcome in newly diagnosed, uniformly treated patients with diffuse large B-cell lymphoma: an explorative pilot study
Source: J Exp Clin Cancer Res. 2018 May 2;37:95. doi: 10.1186/s13046-018-0768-5 (PMC5930939; doi:10.1186/s13046-018-0768-5)

# Supplementary figure 1

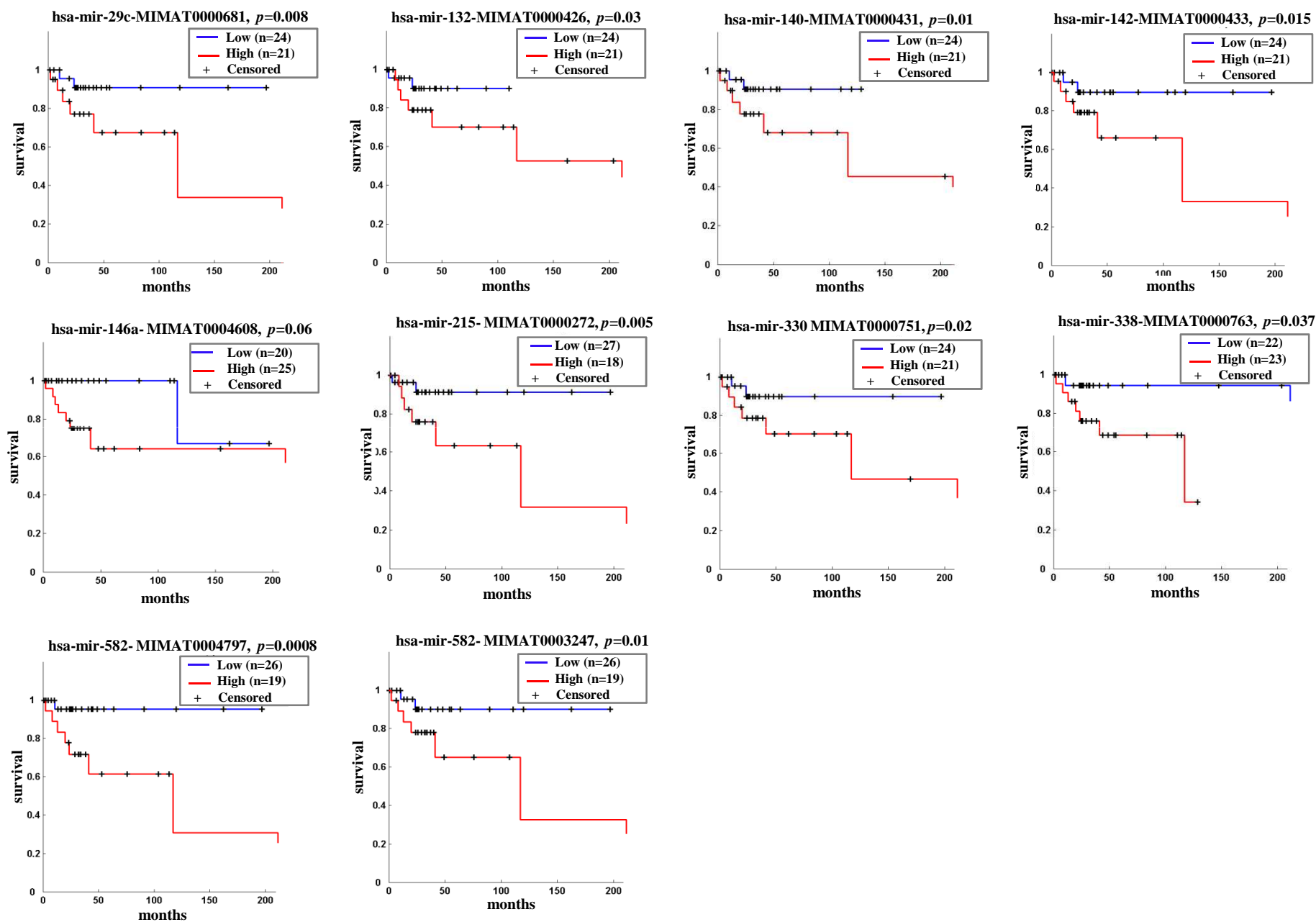

Supplement: Supplementary file 1 — Fig. S1. Kaplan-Meier Overall Survival curves. Correlation between the indicated miRNAs and the Overall Survival of 47 DLBCL patients from TCGA data analysis. (PDF 204 kb) [file 13046_2018_768_MOESM1_ESM.pdf]
